# Oncolytic Viruses as Reliable Adjuvants in CAR-T Cell Therapy for Solid Tumors

**DOI:** 10.3390/ijms252011127

**Published:** 2024-10-16

**Authors:** Ruxandra Ilinca Stilpeanu, Bianca Stefania Secara, Mircea Cretu-Stancu, Octavian Bucur

**Affiliations:** 1 Faculty of Medicine, Carol Davila University of Medicine and Pharmacy, 020021 Bucharest, Romaniabianca.secara@stud.umfcd.ro (B.S.S.); 2 Genomics Research and Development Institute, 020021 Bucharest, Romania; 3 Viron Molecular Medicine Institute, Boston, MA 02108, USA

**Keywords:** oncolytic virus, chimeric antigen receptor T cell, CAR T cell, genetically engineered T cell, immunotherapy

## Abstract

Although impactful scientific advancements have recently been made in cancer therapy, there remains an opportunity for future improvements. Immunotherapy is perhaps one of the most cutting-edge categories of therapies demonstrating potential in the clinical setting. Genetically engineered T cells express chimeric antigen receptors (CARs), which can detect signals expressed by the molecules present on the surface of cancer cells, also called tumor-associated antigens (TAAs). Their effectiveness has been extensively demonstrated in hematological cancers; therefore, these results can establish the groundwork for their applications on a wide range of requirements. However, the application of CAR-T cell technology for solid tumors has several challenges, such as the existence of an immune-suppressing tumor microenvironment and/or inadequate tumor infiltration. Consequently, combining therapies such as CAR-T cell technology with other approaches has been proposed. The effectiveness of combining CAR-T cell with oncolytic virus therapy, with either genetically altered or naturally occurring viruses, to target tumor cells is currently under investigation, with several clinical trials being conducted. This narrative review summarizes the current advancements, opportunities, benefits, and limitations in using each therapy alone and their combination. The use of oncolytic viruses offers an opportunity to address the existing challenges of CAR-T cell therapy, which appear in the process of trying to overcome solid tumors, through the combination of their strengths. Additionally, utilizing oncolytic viruses allows researchers to modify the virus, thus enabling the targeted delivery of specific therapeutic agents within the tumor environment. This, in turn, can potentially enhance the cytotoxic effect and therapeutic potential of CAR-T cell technology on solid malignancies, with impactful results in the clinical setting.

## 1. Introduction

The rising incidence and mortality rates observed annually [1] remain firm arguments for the ongoing prevalence of cancer. The emergence of targeted therapies in cancer molecular medicine and immuno-oncology in recent years has revolutionized the field of cancer treatment [2]. The use of anti-tumor immunotherapy has marked a significant breakthrough in the process of treating cancer by modifying the immune system to improve its capacity to identify and eliminate malignant cells [3].

Chimeric antigen receptor T cell (CAR-T cell) therapy and oncolytic viruses represent novel cancer treatments offering fewer complications compared to traditional chemotherapy and radiotherapy, thus significantly improving the patient’s quality of life [4]. Oncolytic viruses exhibit high selectivity, replicating solely within cancerous cells, effectively destroying them while sparing normal tissues. Meanwhile, in CAR-T cell therapy, T cells are genetically modified and engineered to target specific tumor cell antigens before being retransferred into the patient’s body to combat malignancies. Combining oncolytic viruses with CAR-T cell therapy has the potential to improve the effectiveness of CAR-T cell therapy in destroying solid tumors by increasing the tumor cell permeability for T cells, hence reducing immune system interference.

## 2. Therapeutic Potential of CAR-T Cells in Solid Tumors

### 2.1. Current Status of CAR-T Cell Therapy

**Figure 1:**
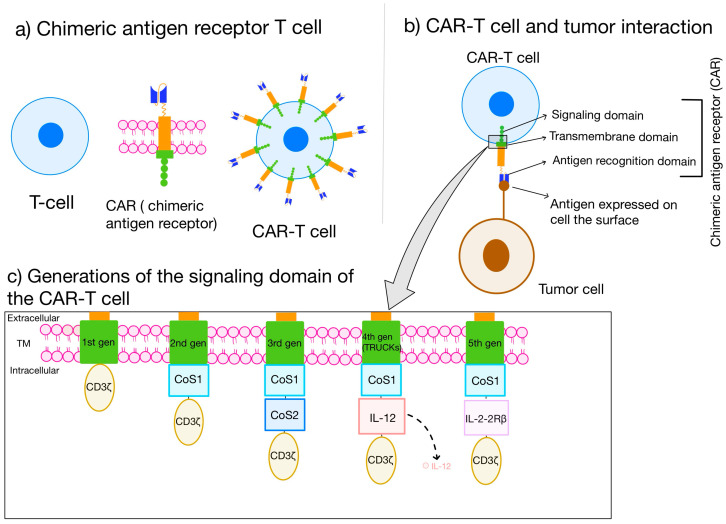
The structure of a CAR-T Cell. (**a**) The T cell, the chimeric antigen receptor (CAR), and the genetically engineered CAR-T cell are illustrated. (**b**) The structure of the CAR-T cell and its interaction with the surface antigen of the tumor cell are presented. (**c**) Differences between the intracellular signaling domains of the 5 generations of CARs. The first generation presents a CD3ζ-derived signaling module. The second generation of CARs is the first to contain a co-stimulatory domain. Co-stimulatory molecules include CD28, 4-1BB (CD137), CD27, and OX40 (CD134). TM—transmembrane domain; TRUCK—T cell redirected for universal cytokine-mediated killing; CoS1,2—co-stimulatory domain; IL-2Rβ—IL-2 receptor β. This figure was created with GoodNotes and adapted from reference [13] for (**a**) and (**b**) and reference [14] for (**c**).

Over the past ten years, there has been a rise in the use of the CAR-T cell therapy as a potential method of immunotherapy for combatting cancer [2,5]. Genetically engineered T cells, which are illustrated in Figure 1, express chimeric antigen receptors (CARs) targeted to cancer cells and can identify tumor-associated antigens (TAAs) located on the surface of cancerous cells irrespective of their expression of the major histocompatibility complex [6]. This recognition process leads to the destruction of the cancerous cells that are targeted through lysis [7]. The mechanism responsible for this phenomenon involves the use of a CAR, consisting of three main parts, which can be observed in Figure 1 [8,9]. By incorporating co-stimulatory or targeting domains, new CAR constructs are continually evolving [10,11,12].

As mentioned before, CAR-T cells are a form of genetically engineered autologous immunotherapy. The process initiates with the patient’s own T cells, which are collected from the bloodstream and modified ex vivo to express a tumor antigen-specific CAR, which includes the antigen-binding domain from a B cell receptor fused to the intracellular domain of a CD3 T cell receptor (CD3-zeta). After being expanded ex vivo, the CAR-T cells are reinfused into the patient. This therapy enables the T cells to recognize specific tumor or cell surface antigens and activate a T cell response independently of MHC recognition. As shown in Figure 1, through the development of five generations of CAR-T cells, various enhancements were introduced to boost their efficacy, such as co-expressing intracellular costimulatory domains like CD28 or 4-1BB (CD137), or pro-effector cytokines like IL-12 were incorporated [13,15].

CAR T cells targeting CD19, CD20, and CD22 antigen-binding domains have been tested in humans, with CD19 being a key biomarker of the B cell lineage [14]. So far, the CAR-T therapy that has achieved the highest level of success involves the targeting of CD19, which is exclusively expressed on both normal and malignant B cells [10]. Since 2017, six CAR-T cell therapies have been approved by the U.S. FDA for hematologic malignancies, which are presented in Supplementary Table S1.

The first ones, approved in 2017, are represented by KYMRIAH^®^ (*tisagenlecleucel*), which was FDA-approved for the treatment of B cell acute lymphoblastic leukemia (B-ALL), and YESCARTA^®^ (axicabtagene ciloleucel), which was approved for treating large B cell lymphoma (LBCL). Subsequently, TECARTUS^®^ (*brexucabtagene autoleucel*) was developed for mantle cell lymphoma in 2020. In the following year, FDA endorsed BREYANZI ^®^ (*lisocabtagene maraleucel*) for treating relapsed or refractory LBCL.

These previously mentioned therapies are designed as anti-CD19 T cells and have proven to be effective in treating B cell malignancies, such as B-ALL and LBCL. In comparison, the two newest CAR-T cell products target the B cell maturation antigen (BCMA). The first one, ABECMA^®^ (*idecabtagene vicleucel*), was approved in 2021, and the second one, CARVYKTI^®^ (*ciltacabtagene autoleucel*), in 2022 [12,16].

In February 2021, the anti-CD19 CAR therapy ARI-0001, classified as an ‘Advanced Therapy Medicinal Product’ (ATMP), received authorization from the Spanish Agency of Medicines and Medical Devices under a ‘hospital exemption’ for treating adult patients (>25 years old) with relapsed/refractory (R/R) B-ALL [17]. This marks a significant milestone as it is the first CAR therapy fully developed within the EU and the first to be approved by a governmental drug agency outside of the centralized marketing authorization process [18].

To improve patient outcomes, scientists are currently focusing on identifying elements that influence the efficacy of CAR-T cells. These factors include the histologic basis of the disease, the lymphodepleting regimen employed, and the specific costimulatory domain integrated into the construct of CAR-T cells [19].

Therefore, the efficacy of this therapy in treating solid tumors is yet to be conclusively established as it faces several challenges, such as inadequate tumor infiltration and trafficking, the existence of an immune-suppressing tumor microenvironment, and adverse reactions that follow the therapy.

### 2.2. Clinical Trials of CAR-T Cell Therapy in Solid Tumors

More than 800 clinical trials have been conducted to investigate the utilization of CAR-T cells in cancer therapy, particularly hematological malignancies [5]. However, progress is still trying to be made in the area of solid tumors [2], as we will further explain.

To begin with, in a phase I-II study (NCT00902044) conducted by Ahmed et al. [20], HER2-CAR-T cells were used in order to treat 19 patients with HER2-positive sarcomas, of which 16 were osteosarcomas, 1 was a primitive neuroectodermal tumor, 1 was Ewing sarcoma, and 1 was a protofibroblastic small round cell tumor. The results consist of a median overall survival (OS) of 10.3 months for the 19 treated patients, but there was also a lack of adverse events even after the administration of a high-dose treatment.

Disialoganglioside (GD2) is highly expressed in neuroblastoma cells, which makes it a potential target for CAR-T cell therapy. A first-in-human phase I clinical trial (NCT04196413) was conducted by Majzner et al., with GD2 CAR-T cells being used to treat four patients with H3K27M-mutated DIPG (diffuse intrinsic pontine glioma) or spinal cord DMG (diffuse midline gliomas) [21]. Among the four patients, three exhibited improvements in both clinical symptoms and radiographic findings along with high levels of pro-inflammatory cytokines in the plasma and cerebrospinal fluid without any on-target/off-tumor (OTOT) toxicity. The OTOT might have resulted from the presence of the targeted antigen on both tumor cells and at least some normal cells.

The epidermal growth factor receptor (EGFR) is essential in the development and progression of various solid tumors and has become a significant target for therapy in different types of cancer, including breast, colorectal, gastroesophageal, and non-small cell lung carcinomas [22]. A phase I clinical study (NCT01869166) examined EGFR CAR-T cell therapy in 11 patients with refractory/relapsed non-small cell lung cancer (NSCLC) that was EGFR positive. The study demonstrated that two patients achieved a partial response (PR), and five others obtained stable disease (SD) for 2 to 8 months without experiencing severe toxicity [23].

Elevated levels of the carcinoembryonic antigen (CEA) have been linked to an unfavorable cancer prognosis [24], prompting investigations into CEA-targeted therapies for lung [25], breast, pancreatic, and gastric cancers [26,27], especially colorectal cancer (CRC) [28]. In a phase I trial with doses that have been progressively raised (NCT02349724), CAR-T cell therapy targeting CEA expressed in metastatic CRC was found to be well tolerated and showed promising clinical outcomes. Specifically, 7 out of 10 patients demonstrated SD for a maximum of 30 weeks, while 2 patients experienced tumor reduction with no reported adverse events [29].

### 2.3. Opportunities and Future Prospects in CAR-T Cell Therapy

The unique characteristics specific to solid tumors and the inherent limitations of the CAR-T cell therapy approach make it a challenging application [30,31,32]. In cases where engineered T cells designed to target specific TAAs are used to treat cancer, there is a possibility of antigen escape. This occurs when the tumor cells, under the influence of selection pressure, start favoring the growth of cells that do not possess the targeted antigens [33]. In addition to the aforementioned phenomenon, tumor cells can downregulate the expression of an antigen, and as CAR-T cells can be sensitive to antigen density, the low-level expression of tumor antigens can also result in escape. This leads to the development of dual CAR constructs or tandem CARs, which represent a single CAR structure with two single-chain variable fragments [34].

Secondly, CAR-T cells may target TAAs shared by tumors and normal tissue due to the narrowed availability of tumor-specific antigens, which consequently results in on-target/off-tumor toxicity [35]. This phenomenon could be exemplified by patients from a clinical trial who experienced cardiogenic shock due to the cross-reaction between the TCR of the T cell infusion they received and their myocardial protein, Titin [36]. What needs to be considered is whether the normal cell can be depleted with acceptable toxicity, such as CD19 CAR-T cell therapy, which results in B cell aplasia [37], or whether expression is drastically higher on tumor cells, thus favoring tumor cell killing with limited healthy tissue OTOT, such as GD2 CAR-T cells [38].

**Figure 2:**
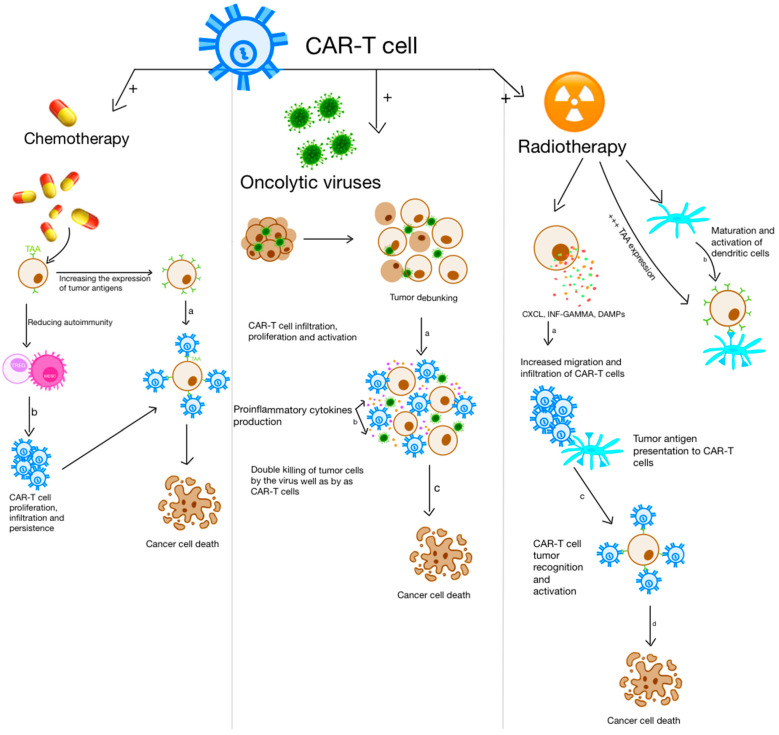
Three possible combinations of CAR-T cell therapy with other therapies are presented. From right to left: (**a**) chemotherapy, (**b**) oncolytic viral therapy, and (**c**) radiation therapy. This figure was created with GoodNotes and adapted from reference [2].

Numerous approaches have been devised to address these hurdles and enhance the effectiveness of CAR-T cells in the context of solid tumors. These include optimizing CAR constructs, identifying innovative therapeutic combinations, and modulating inhibitory conditions, thereby enhancing the specificity, infiltration, and effectiveness of the CAR-T cell treatment [2]. Chemotherapy and radiotherapy are the conventional choices for combination with CAR-T therapy, as shown in Figure 2 and exemplified further.

As a conditioning regimen, the most used chemotherapy drugs in CAR-T cell therapy are cyclophosphamide and fludarabine, which further lead to a process of lymphodepletion through which a better expansion of T cells is achieved [39,40].

Chemotherapy can inhibit suppressive immune cells, leading to the increased persistence of CAR-T cells, and sensitize tumor cells to CAR-T cell activity by enabling granzyme B penetration into tumor cells [41,42,43]. The positive effects of combining chemotherapy with CAR-T cell therapy have been reviewed in detail by Safarzadeh Kozani P et al. [44]. By employing this combination therapy, it becomes possible to tackle the problem of CAR T cells’ migration to the TME, ultimately leading to a more potent and efficient outcome, which translates into higher survival rates [45].

Radiation therapy can eliminate cancer cells by triggering apoptotic and necrotic processes, leading to dendritic cell maturation and activation, and stimulating the presentation of tumor antigens [46]. Furthermore, radiation releases damage-associated molecular patterns (DAMPs) and IFN-γ, which promote the infiltration and migration of CAR-T cells into the tumor [47]. Combining radiotherapy with CAR-T cell therapy has been reported to improve the anti-tumor efficacy in a synergistic manner [44,48] in solid tumors such as glioblastoma.

## 3. Therapeutic Potential of Oncolytic Viruses in Solid Tumors

### 3.1. Current Status of Oncolytic Virus Therapy

Viruses are infectious particles that rely on host cells for their survival and proliferation, causing pathogenesis and inflammation within them [49]. Oncolytic viruses are naturally occurring or are genetically modified to be replication-competent [6]. These viruses have the ability to selectively infect and/or replicate in cancer cells, leading to their preferential destruction [50,51,52]. Typically, oncolytic viruses achieve their specificity by relying on abnormalities in cell surface receptors or alterations in gene expression within malignancies that occur during tumor progression [53].

The cancer-targeting mechanisms of viruses can generally be achieved through two main strategies: deleting viral genes necessary for replication in normal cells but not in tumor cells and utilizing tissue- or tumor-specific promoters to regulate critical viral genes. Specific tumor targeting can also be accomplished by focusing on various molecular regulators or steps of the cell cycle [53,54].

Oncolytic viruses can be categorized as either RNA or DNA viruses based on the content of their genome, which is packaged inside a protein coat called the capsid [55,56]. DNA oncolytic viruses are preferred due to their larger genome, the high stability of their polymerase enzyme, and their high proliferative ability [50]. RNA viruses, on the other hand, are more suitable for tumors in the central nervous system due to their smaller size and ability to cross the blood–brain barrier [57].

Regarding methods of administration, multiple routes of delivery have been developed due to the major importance of an optimal delivery in the success of the treatment [58]. Intratumoral administration stands as the main method, and it enables precise control over the concentration of the virus at the intended site while minimizing the risk of unintended effects on other organs. However, it is worth noting that intratumoral delivery is much more applicable to superficial tumors like melanoma than to deep-seated tumors such as glioblastoma. On the other hand, intravenous administration represents a simpler method of delivery, especially during the clinical trial phase, which numerous researchers lean toward given the possible obstacles that can arise during intratumoral delivery, for example, in metastasis.

Many oncolytic viruses function like vaccines, inducing strong and specific anti-tumor responses [4] which are mediated by TCD8+ and the formation of significant memory cells [59,60]. Therefore, it could be argued that oncolytic viruses not only have oncolytic activity, but they also have the ability to trigger inflammation and stimulate immune responses against both themselves and tumor cells. The outcome of the immune response is complex, and the anti-tumor immunity mediated by oncolytic viruses can be extensively effective [61,62].

Moreover, they have low toxicity levels, as shown in preclinical toxicology studies [63], and a tolerable safety profile that is largely different from that of other cancer treatments [64]. Globally, only four oncolytic viruses and one non-oncolytic virus have been approved for cancer treatment, with talimogene laherparepvec (T-VEC) being the only widely accepted therapy [64]. T-VEC is used for patients with melanoma that has recurred following initial surgery [4] and was first authorized in 2015 [65].

### 3.2. Clinical Trials for Oncolytic Virus-Related Therapy

In the past two decades, >3000 patients enrolled in clinical trials using oncolytic viruses, including adenovirus, herpesvirus, picornavirus, measles virus, vaccinia virus, reovirus, poliovirus, coxsackievirus, vesicular stomatitis virus, parvovirus, and retrovirus, which were studied in >95 different studies [66]. However, as previously mentioned, only four oncolytic viruses have been approved for advanced solid tumor treatment so far.

To begin with, a non-enveloped RNA virus was derived from the native ECHO-7 strain of a picornavirus. It acts as an immunomodulator with anti-tumor effect, and it is approved for the treatment of melanoma, the local treatment of skin and subcutaneous metastases of melanoma, and for the prevention of relapse and metastasis after radical surgery in Latvia, Georgia, Armenia, and Uzbekistan [67].

One year later, H101, a modified adenovirus, was approved by the Chinese State Food and Drug Administration to be used alongside cytotoxic chemotherapy as a treatment for patients with late-stage refractory nasopharyngeal cancer [68]. In the United States, H101 is currently part of an undergoing clinical trial [69]. In 2015, T-VEC, an attenuated herpes simplex virus type 1 (HSV-1; DNA virus, enveloped)-expressing granulocyte-macrophage colony-stimulating factor, was approved by the U.S. Food and Drug Administration for the local non-surgical management of unremovable skin, underlying tissue and lymph node in patients with recurrent melanoma after initial surgery [70]. In addition, G47D, a third-generation oncolytic HSV-1, was recently approved in 2021 in Japan for treating the recurrence of glioblastoma [71].

### 3.3. Opportunities and Future Prospects

An initial surge of hope arose in the 1990s when it was thought that oncolytic viruses could trigger a domino effect throughout the entire tumor, resulting in the eradication of cancer [6,52]. However, clinical experience showed that patient outcomes were not as successful as what was observed in cultured cells or immune-competent murine models [51]. Barriers such as the viral load, spread, and antiviral immunity hinder the introduction of oncolytic virotherapy into clinical practice [72].

Researchers have tried to promote viral spread within tumor masses by arming oncolytic viruses with enzymes to break down extracellular matrix proteins [72]. In the past twenty years, accumulated evidence has shown that, while antiviral immunity may impede the sequential progression of oncolytic viruses, the direct destruction of cancer cells by oncolytic viruses triggers a robust immune response against tumors, which plays a vital role in their effectiveness [50,51,55,73].

The process of viral infection and replication induces tumor necrosis, subsequently attracting immune cells to the tumor site. This event initiates an innate immune response and promotes the development of adoptive immunity against cancer-specific neoantigens [52]. In order to augment the anti-tumor immune response induced by oncolytic viruses within solid tumors and counteract the immunosuppressive TME, scientists are actively exploring approaches to combine oncolytic viruses with immune modulators like cytokines, immune checkpoint inhibitors, or immune costimulators [52].

In line with this, a growing body of research has explored the use of oncolytic viruses in hematological malignancies, showing promising progress. Several phase I clinical trials are currently underway, evaluating oncolytic reovirus in combination with lenalidomide or programmed death 1 (PD-1) immune checkpoint inhibitors for multiple myeloma [74,75,76].

## 4. Oncolytic Viruses as Reliable Adjuvants in CAR-T Cell Therapy

While CAR-T cell therapy has shown promising results in treating hematological malignancies, the possibility of it being efficient in treating solid tumors is limited, indicating the need to search for combined targeted therapies to achieve complete responses [77]. Oncolytic viruses can potentially assist CAR-T cells in overcoming a few of the challenges associated with solid tumors [50]. By inserting oncolytic viruses into target cells, they can alter the tumor microenvironment from an immunosuppressive environment to an immunostimulatory one [2]. This modification, illustrated in Figure 2, can turn cancer cells into cytokine (IFNβ, IL-1β, IL-12, IL-4, and TNFa) and chemokine (CXCL9, CCL5, and CX3CL1) factories, thus facilitating the recruitment and activation of immune cells, with their main representants being CAR-T cells and factors [78]. The stimulation of CD8+ T cells targeting pathogens or tumors relies on three fundamental signals: TCR engagement (signal 1), co-stimulation (signal 2), and an inflammatory trigger (signal 3). Signal 3 is commonly induced by cytokines like interleukin (IL)-12 or type I IFNs. Second- or third-generation CARs engaging TAAs equip engineered T cells with signals 1 and 2 [79].

### 4.1. Preclinical Studies of CAR-T Combined with OV

Cancer cells grow and proliferate more rapidly than the immune system can manage, using strategies that evade detection and prevent immune cell attacks. From the onset of cancer, these cells begin constructing a tumor microenvironment (TME), and by the time a detectable mass forms, the TME is usually well established. This makes it challenging for the immune system to effectively eliminate cancer cells [80].

However, the use of oncolytic viruses can counteract this inhibition. An extensive evaluation has been conducted on genetically modified oncolytic viruses in both pre-clinical and clinical investigations, demonstrating encouraging outcomes, particularly in animal studies that explored the combination of oncolytic viruses with CAR-T cell therapy.

Most studies that combine CAR-T cell therapy with oncolytic viruses are conducted at the preclinical stage, and since they represent the focus of this review, they are presented in Table 1. These studies consistently exhibit therapeutic advantages in mice when oncolytic viruses are combined with CAR-T cells, such as diminished tumor growth and metastasis, along with enhanced survival rates [6]. Typically, these studies employ xenograft immunodeficient mice models engrafted with human CAR-T cells. Oncolytic viruses are commonly administered through intratumoral injection to draw intravenously administered CAR-T cells toward the virus-activated TME, thereby enhancing the process of oncolysis [6].

Preclinical investigations involving animals have shown the effectiveness of combining oncolytic viruses with CAR-T cell therapy in treating various forms of carcinomas, including abdominal ones, but also head and neck carcinoma and neuroblastoma.

To begin with, in 2018, Anna Wing et al. conducted a study on pancreatic ductal carcinoma/colorectal carcinoma, where they used Onc. Ad-EGFR BiTE, which is an oncolytic adenovirus equipped with an EGFR-targeting, bispecific T cell engager. It was discovered that the simultaneous application of this modified virus alongside CAR-T cells, which were engineered to directly target the folate receptor alpha (FR) antigen, can enhance the overall efficacy of CAR-T cells in treating solid tumors. This improvement occurs due to the BiTE molecule secretion, originating in the oncolytic adenovirus, of the cancer cells that have been contaminated. Although tumor cells lack CAR-specific antigens (FR-α in this case), they can still be targeted and destroyed by CAR-T cells with the help of the aforementioned BiTE secretion [81]. Therefore, tumor heterogeneity can become an accessible target for treatment.

Moreover, using the Onc. Ad5Δ24 virus (80), which is armed with the RANTES (regulated upon activation, normal t cell expressed and presumably secreted) chemokine, secreted by platelets which have been activated predominantly during flow conditions and IL-15, along with Ganglioside GD2-specific CAR-T cells has been proven to enhance the attraction and survival of the CAR-T cells in the neuroblastoma tumor environment [82]. Firstly, what augmented the overall survival rates in the animal models with tumors was that the oncolytic virus stimulated the caspase pathways in tumor cells which were revealed to CAR-T cells. What further attracted CAR-T cells at the level of tumors were the increasing intratumoral levels of IL-15 and RANTES. Thus, their combined treatment led to improved treatment outcomes in solid tumors in cancer-bearing mice models.

**Table 1:** Preclinical studies of the effectiveness of combining CAR-T cell therapy and oncolytic viral therapy in solid tumors from 2022 to 2018.

Type of Solid Tumor (Starting with Most Recent Ones)	Publication Year and Author	CAR-T Cell Targets	CAR-T Cell Generation/CAR Components	Oncolytic Agents	Type of Virus
Glioblastoma [83,84]	2022; Zhu, G.	GBM cells	lentiviral vector containing a trCD27 (including extracellular portion of CD27, aa1-191), CD8 transmembrane domain, and a CD137 costimulatory domain and CD3ζ	oHSV-1	HSV-1
	2022; Chalise, L.	PDPN-expressing glioma cells	lentiviral vector linked with the EF1α promoter, leader sequence- and Lp2-based scFvs, CD28, 4-1BB, and CD3ζ (third-generation CAR)	G47D	Third-generation recombinant HSV-1
Subcutaneous melanoma or intracranial glioma tumor [85]	2022; Evgin, L.	subcutaneous B16EGFRvIII tumors	EGFRvIII	intratumoral oncolytic VSV expressing mIFNβ	VSV
Solid tumor [86]	2021; Chen, T.	CD19	NCI-H292-CD19 cell line generated through lentivirus-mediated transduction of T cells isolated from healthy human PBMCs	rTTVDTK armed rationally with IL-21	VV
Glioblastoma [87]	2021; Huang, J.	glioblastoma cells	B7H3-targeted CAR-T	oAD-IL7	Adenovirus
Pancreatic adenocarcinoma [77]	2021; Shaw, A.R.	CD44v6+ cancer cell lines	HER2-specific CAR T cells	CAdTrio (oncolytic-helper binary adenovirus encoding an IL-12 and PD-L1Ab)	Adenovirus
B cell lymphoma [88]	2021; Wenthe, J.	CD19+ B cell lymphoma lines	Third-generation CAR containing both CD28 and 4-1BB co-stimulatory domains	LOAd703—delolimogene mupadenorepvec	Adenovirus
Liver cancer or hepatocellular carcinoma [89]	2020; Tang, X.	CD19+	HER2-specific scFv fused to an IL-13Rα2-binding IL-13 mutein as a tandem CAR exodomain	AdC68-TMC-Tcd19	Adenovirus
Melanoma [90]	2020; Aalipour, A.	CD19+	second-generation murine mCD19 CAR T cells containing CD3ζ and CD28 costimulatory domains	mCD19VV	VV
Breast cancer [91]	2020; Li, Y.	mesothelin	meso-CAR-T	Oads targeting transforming growth factor β signaling (rAd.sT)	Adenovirus
Pancreatic adenocarcinoma or squamous cell carcinoma [92]	2020; Porter, C.E.	CD44v6+ cancer cell lines	HER2-specific CAR T cells	CadTrio	Adenovirus
Breast cancer [93]	2019; Park, A.K.	CD19	CD19-CAR T cells	oncolytic vaccinia virus coding for CD19t (OV19t)	VV
Lung cancer [94]	2018; Moon, E.K.	mesothelin	modified chimeric antigen receptor (CAR)-transduced human T cells to deliver CXCL11 (CAR/CXCL11)	modified oncolytic vaccinia virus (VV) engineered to produce CXCL11 (VV.CXCL11)	VV
Pancreatic ductal carcinoma or colorectal carcinoma [81]	2018; Wing, A.	FR-α	anti-CD3 single-chain variable fragment (scFv) fused to an anti-tumor-associated antigen scFv via a flexible linker	oncolytic adenovirus armed with an EGFR-targeting, bispecific T cell engager (Oad-BiTE)	Adenovirus

Oncolytic viruses have the capacity to work with lymphocytes against cancerous growths [4]. However, since these viruses can prompt an immune reaction, the therapeutic doses required to overcome the immune response, including antibodies, are very high [95,96]. To address this problem, Heather VanSeggelen et al. used CAR-T cells loaded with low-dose oncolytic viruses, such as modified VSV and vaccinia virus. Their study determined that there was no difference in the CAR expression levels in virus-loaded CAR-T cells and intact CAR-T cells, and the function of the modulated CAR-T cells was not disrupted [97]. These results suggest that ‘variable oncolytic virus-loaded CAR-T cells’ can be included in future pre-clinical research to evaluate the effectiveness of this novel approach in animal models of cancer.

### 4.2. Delivery of Oncolytic Virotherapy Using CAR-T Cells

Oncolytic herpes simplex virus strains have the potential to trigger immune-stimulatory responses within the tumor microenvironment. However, the traditional intravenous administration of these oncolytic viruses has not yet succeeded in achieving sufficient viral accumulation at tumor sites [72].

A major obstacle to the widespread use of these viral agents in treating metastatic conditions lies in the challenge of delivering them systemically to well-established tumors that cannot be directly injected. This challenge is partly due to viral neutralization, the nonspecific binding of virus particles to various host cell types, and the active sequestration of particles.

In the support of this matter, a recent study utilized CAR-T cells as carriers to deliver the myxoma oncolytic virus (MYXV), with the T cells being pre-infected ex vivo through a spin infection protocol. Their results indicate a potential 7-day time frame for MYXV-infected CAR-T cells to deliver the virus into tumor cells [98].

Furthermore, recently, Zhang et al. successfully assessed the CAR-T cell delivery of an engineered oncolytic HSV to solid tumors using both immunodeficient and immunocompetent orthotopic GBM mouse models [99]. Unexpectedly, both in vitro and in vivo studies demonstrated the efficient delivery of the oHSV by B7-H3 CAR-T cells into the corresponding tumor cells, leading to tumor suppression. Their study revealed, through the tumor suppression achieved both in vitro and in vivo, the potential of this delivery method to be translated into clinical practice for solid tumors characterized by widespread metastasis and effective targeting by B7-H3 CAR-T cells.

### 4.3. Prospects and Obstacles Associated with Integrating Adoptive Cellular Therapy with Oncolytic Virotherapy

Both the EMA and U.S. FDA have a major requirement regarding extensive preclinical data, encompassing studies using relevant animal models, to support the rationale for combining therapies; the regulatory stance of the EMA and U.S. FDA would be to guarantee that any combined therapy meets strict standards for safety, efficacy, and quality before it can be approved for clinical use. There is not a specific guideline or stance from the EMA solely dedicated to the combination of virus cell therapy and CAR T cell therapy [100]. Nonetheless, both virus cell therapy (using oncolytic viruses, for example) and CAR T cell therapy are emerging fields in cancer treatment, and their combination holds promise in enhancing the efficacy of immunotherapy.

Owing to tumor heterogeneity, pre-existing immunity in the patient’s body, and the lack of effective penetration into tumor tissue, the efficacy of an oncolytic virus as single-agent therapy is limited. Hence, in order to enhance treatment efficacy and foster synergistic effects, researchers utilize combination therapies incorporating oncolytic viruses alongside other anti-tumor modalities [101].

Identifying an optimal combination hinges upon a comprehensive grasp of the immune system, advanced bioinformatic analyses, predictive models, and a diverse array of thoroughly characterized treatment modalities. In essence, the intricate workings of oncolytic viruses facilitate debulking of the tumor, the liberation of tumor-specific neoantigens, and the induction of inflammatory alterations within the TME. Henceforth, the concept of integrating ACT approaches with oncolytic virus therapy has emerged as a rational strategy to potentially amplify the infiltration and expansion of adoptively transferred cells through pro-inflammatory signaling mediated by the virus infection [102].

With the recent endorsement of CAR T cell therapies targeting CD19, the hypothesis of adoptive T cell therapy for cancer is the cutting edge of the immuno-oncology field. However, despite the promising outcomes observed in specific patient subgroups with these therapies, a limitation of this approach lies in its dependence on the identification of appropriate TAAs and neoantigens for targeting.

As previously stated, CD19 CAR-T cells have shown remarkable efficacy in treating hematological malignancies, particularly B-ALL [103]. Notwithstanding, those same achievements have not been effectively applied to other types of lymphomas and leukemias or solid tumors.

Apart from antigen escape, the mechanisms underlying CART-T cell failure can obviously be due to the lack of T cell persistence and T cell dysfunction or exhaustion. Consequently, a large number of strategies have been proposed to enhance CAR-T cells’ effectiveness by improving tumor infiltration, persistence, or overall function. Chemotherapy and radiotherapy build the foundation of cancer treatment and, in combination with CAR-T cells, can highly adjust the TME to eliminate immunosuppressive cells and enhance T cell persistence. Recent clinical efforts have focused on combining CAR-T cells with immune checkpoint blockade (ICB) to reduce T cell exhaustion and thereby enhance CAR-T cell efficacy within the hostile TME [103]. Additionally, considering the fact that each tumor presents a unique genetic profile, targeted therapies frequently require costly and time-intensive molecular screening of tumor biopsies, followed by the production of personalized treatments [102].

Although OV therapy holds promise in augmenting ACT, various challenges hinder the extent to which OVs can optimize therapeutic outcomes in combination therapy. While extensive research has been conducted to delineate the strengths of Ovs and ACTs against cancers, recent studies have begun to explore the synergistic potential between these two innovative approaches, aiming to enhance therapeutic efficacy beyond what either approach can achieve individually. Despite the nascent stage of the field, efforts to maximize the potential of such combinations are already underway. Research on this combination is in its preliminary stages, with numerous preclinical investigations being documented, which are further explained in Table 2, and four phase I clinical trials being initiated, as seen in Table 3.

In summary, the targeted depletion or functional modulation of such cells holds promise for enhancing the anti-tumor response in the context of OV + ACT.

**Table 2:** A list of selected preclinical studies investigating the combined use of OV and adoptive cell therapy.

Oncolytic Virus Type	Transgenes	CAR-T Cells Involved	Type of Cancer	References for the Studies
Vaccinia virus (vvDD)	CXCL-11	mesothelin-CAR-T	lung cancer	Moon (2018) [94]
Chimeric vaccinia virus (CF33)	truncated CD19	CD19-CAR	breast cancer	Park (2019) [93,104]
Vesicular stomatitis virus	mIFNβ	mEGFRvIII	murine melanoma	Evgin (2020) [105]
Vaccinia Western Reserve	CCL5	CCR5-NK	various carcinomas	Li (2020) [106]
Adenovirus (Ad5/3Delta24)	CD44v6xCD3 bispecific, IL-12, anti-PDL1 expressed by co-injected helper Ad	HER2-CAR-T	pancreatic ductal adenocarcinoma, squamous cell carcinoma	Porter (2020) [92]
Adenovirus (rAd.sT)	TGFb decoy	mesothelin-CAR-T	breast cancer	Li (2020) [91]
Vaccinia virus	CD19	CD19-CAR-T	melanoma	Aalipour (2020) [90]
Adenovirus	CD19 tag	CD19-CAR-T	liver cancer	Tang (2020) [89]
Adenovirus (Ad5/3Delta24)	IL12, anti-PDL1 expressed by co-injected helper Ad	HER2-CAR-T	pancreatic cancer	Rosewell Shaw (2021) [107]
Vesicular stomatitis virus (VSVDeltaM51)	IL-15	NKT	pancreatic cancer	Nelson (2022) [108]
HSV-1 (G47Δ)	N/A	Lp2-CAR-T	glioblastoma	Chalise (2022) [83]
Vesicular stomatitis virus, reovirus	mIFNβ (VSV)	mEGFRvIII	murine melanoma, glioma	Evgin (2022) [3,85]
HSV-1	OX40L, IL-12	TILs	colon cancer, pancreatic cancer	Ye (2022) [109]

**Table 3:** Clinical trials combining OV and adoptive cell therapy to treat cancers. Data are available at https://www.clinicaltrials.gov/; accessed on 25 September 2024.

Biological Agent	Combination	Indication	Status	Sponsor	Trial ID
TILT-123 (adenovirus codin TNFa and IL2)	adoptive cell therapy with TILs	metastatic melanoma	recruiting, phase I	TILT Biotherapeutics Ltd.	NCT04217473 [110]
CAdVEC (binary oncolytic adenovirus)	HER2-specific CAR-T cells	advanced solid tumors	recruiting, phase I	Baylor College of Medicine	NCT03740256
VCN-01 (oncolytic adenovirus expressing hylauronidase)	mesothelin-specific CAR-T	pancreatic cancer, serous ovarian cancer	recruiting, phase I	University of Pennsylvania	NCT05057715

## 5. Benefits, Limitations, and Future Prospects

**Figure 3:**
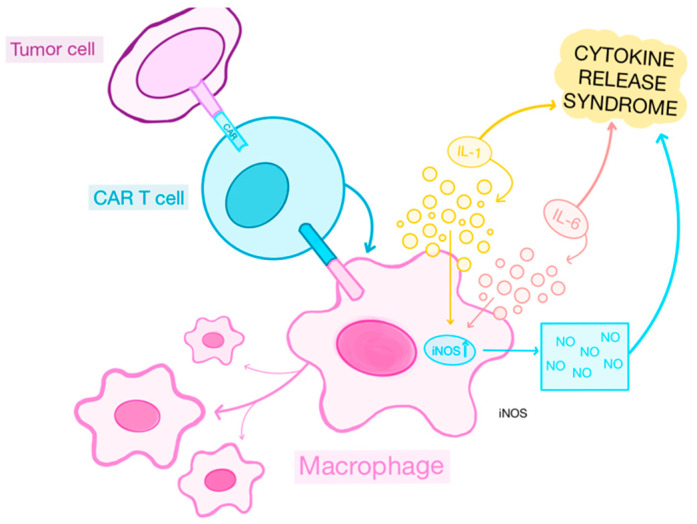
The effect of CAR-T cells on macrophages and the development of cytokine release syndrome. The interaction of macrophages with activated CAR-T cells further leads to the release of chemokines and cytokines such as IL-1, IL-6, and iNOS, causing supplementary inflammatory reactions, resulting in CRS. This figure was created with GoodNotes and adapted from reference [117].

Drawing upon numerous preclinical and clinical studies investigating CAR-T cell therapy and oncolytic virotherapy [95,96,97], it is reasonable to anticipate that the combination of these two interventions would yield enhanced efficacy in the treatment of cancer patients, surpassing the outcomes achieved by either approach alone [6]. Nevertheless, the primary toxicities frequently linked to CAR-T cell therapy encompass CRS, illustrated in Figure 3, and ICANS, which present challenges for its prevalent use in outpatient settings [111]. ICANS can occur either concurrently with or without CRS [111,112]. Patients who manifest CRS present with symptoms such as fever, tachycardia, and hypotension and increased levels of cytokine in their serum, including IFN, IL-6, IL-2, IL-2 receptor, IL-8, and IL-10 [111].

Additional research is required to devise combination therapies to effectively mitigate the exacerbated effects of the cytokine storm demonstrating comparable efficacy to a higher dosage of intravenously infused CAR-T cells while avoiding the induction of elevated cytokine levels in the peripheral blood [21].

Nonetheless, not all virus-induced effects facilitate the activity of CAR-T cells. In the initial phases of viral infection, type I IFNs trigger apoptosis in memory T cells [113,114]. A representative could be an oncolytic VSV that expresses IFNβ, which causes a substantial loss of CAR-T cells, whereas an oncolytic reovirus causes only moderate attrition [85].

Moreover, certain oncolytic viruses, such as oncolytic VSV, vaccinia virus, and Newcastle disease virus, prompt vascular disruption, which intensifies tumor eradication [115,116,117] but may impede the ability of intravenously administered CAR-T cells to reach the intended tumor cells. Therefore, one potential approach to avoid oncolytic virus-mediated conditions resistant to CAR-T cells could be to administer CAR-T cells to the tumor first and then use oncolytic viruses, delivering them intratumorally [6].

## 6. Conclusions

Immunotherapies for cancer utilizing CAR-T cells have shown significant clinical and preclinical outcomes, particularly in hematological malignancies. Progress has been made in this field, and various challenges have been faced as well, as revealed in clinical trials that included patients with solid tumors. Nevertheless, progress in the field of CAR-T cell therapy relies on striking a balance between effective anti-tumor activity and minimizing detrimental side effects. Recent advances, including the approval of six CAR-T cell therapies for hematologic malignancies, and the approval of the first oncolytic virus (talimogene laherparepvec), represent significant progresses in cancer immunotherapy.

Preclinical and in vivo investigations have showcased the fact that combined treatments are more effective than each treatment used alone. To achieve better outcomes with fewer side effects, it is essential to conduct further research on how to balance the dosage, delivery, and timing of CAR-T cells and oncolytic viruses in combination therapy. As progress is being made in advancing these two types of therapy separately, a deeper understanding of their potential synergies will be achieved, and more effective treatment choices for patients with advanced and treatment-resistant solid tumors will be developed.

## Supplementary Materials

The supporting information can be downloaded at https://www.mdpi.com/article/10.3390/ijms252011127/s1.

## Abbreviations

CAR-T cells—chimeric antigen receptor T cell; TAAs—tumor-associated antigens; TAMs—tumor-associated macrophages; MHC-I—major histocompatibility complex I; DNA—deoxyribonucleic acid; RNA—ribonucleic acid; TCD8+—cluster of differentiation 8+ T cells; T-VEC—talimogene laherparepvec; VSV—vesicular stomatitis virus; VV—vaccinia virus; ECHO viruses—enteric cytopathic human orphan; U.S. FDA—United States Food and Drug Administration; HSV—herpes simplex virus; TME—tumoral microenvironment; PD-L1—programmed death-ligand 1; scFv—single-chain fragment variable; B-ALL—B cell acute lymphoblastic leukemia; LBCL—large B cell lymphoma; GD2—disialoganglioside; IL—interleukin; DIPG—diffuse intrinsic pontine glioma; DMG—diffuse midline glioma; OTOT—on-target/off-tumor; HER2—human epidermal growth factor receptor 2; EGFR—epidermal growth factor receptor; NSCLC—non-small cell lung cancer; CEA—carcinoembryonic antigen; CRC—colorectal cancer; DAMPs—damage-associated molecular patterns; IFN—interferon; BiTEs—bispecific T cell engagers; RANTES—regulated upon activation, normal T cell expressed and presumably secreted; FR—folate receptor; CRS—cytokine release syndrome; ICANS—immune effector cell-associated neurotoxicity syndrome; NK—natural killer; iNOS—inducible nitric oxide synthase; EGFRvIII—third-generation anti-epidermal growth factor receptor variant III; mIFNβ—mouse interferon-β; TTVDTK—tumor-targeted replicating vaccinia virus Tian Tan strain with deletion of TK gene; PBMCs—peripheral blood mononuclear cells; ACT—adoptive cellular therapies; EMA—European Medicines Agency; PDPN—podoplanin.
