# Supplementary material for: Oncolytic Viruses as Reliable Adjuvants in CAR-T Cell Therapy for Solid Tumors
Source: Int J Mol Sci. 2024 Oct 16;25(20):11127. doi: 10.3390/ijms252011127 (PMC11508774; doi:10.3390/ijms252011127)
Supplement: Supplementary file 1 [file ijms-25-11127-s001.zip › ijms-3188981-supplementary.pdf]

Oncolytic viruses as reliable adjuvants in CAR-T Cell Therapy for solid tumors

Ruxandra Ilinca Stilpeanu[1], Bianca Stefania Secara[1], Mircea Cretu[2,] *, Octavian Bucur [2,3,] *
[1]Faculty of Medicine, Carol Davila University of Medicine and Pharmacy, Bucharest, Romania
[2]Genomics Research and Development Institute, Bucharest, Romania
[3]Viron Molecular Medicine Institute, Boston, MA 02108, USA

*** Correspondence:**
Octavian Bucur, MD, PhD, General Director, Genomics Research and Development Institute, Bucharest, Romania; Emails: octavian.bucur@genomica.gov.ro; octavian.bucur@gmail.com
Mircea Cretu, PhD, Genomics Research and Development Institute, Bucharest, Romania; Email: mircea.cta@gmail.com

Supplementary Table S1. CD19-directed genetically modified autologous T cell immunotherapies and B cell maturation antigen (BCMA)-directed genetically modified autologous T cell immunotherapies
I. CD19-directed genetically modified autologous T cell immunotherapies that present the following toxicities: cytokine release syndrome and neurological ones; this table was adapted from references[14-19].

Product name and year it was approved	Indications and usage
**1. KYMRIAH®** ***(tisagenlecleucel)***<br><br>**FDA-approved in 2017[14]**<br><br>**Limitations of use:**	• Patients up to 25 years of age with B cell precursor acute lymphoblastic leukemia (ALL) that is refractory or in second or later relapse.<br>• Adult patients with relapsed or refractory (r/r) large B cell lymphoma after two or more lines of systemic therapy, including diffuse large B cell lymphoma (DLBCL) not otherwise specified, high grade B cell lymphoma, and DLBCL arising from follicular lymphoma.<br>• Adult patients with relapsed or refractory follicular lymphoma (FL) after two or more lines of systemic therapy.<br><br>KYMRIAH is **not** indicated for treatment of patients with primary central nervous system lymphoma.
**2. YESCARTA®** **(axicabtagene ciloleucel)**<br><br>**FDA-approved in 2017[15]**<br><br>**Limitations of use:**	• Adult patients with large B cell lymphoma that is refractory to first-line chemoimmunotherapy or that relapses within 12 months of first-line chemoimmunotherapy.<br>• Adult patients with relapsed or refractory large B cell lymphoma after two or more lines of systemic therapy, including diffuse large B cell lymphoma (DLBCL) not otherwise specified, primary mediastinal large B cell lymphoma, high grade B cell lymphoma, and DLBCL arising from follicular lymphoma.<br>• Adult patients with relapsed or refractory follicular lymphoma (FL) after two or more lines of systemic therapy.<br><br>YESCARTA is **not** indicated for treatment of patients with primary central nervous system lymphoma.

**3. TECARTUS®** ***(brexucabtagene autoleucel)***<br><br>**FDA-approved in 2020[16]**	• Adult patients with relapsed or refractory mantle cell lymphoma (MCL).<br>• Adult patients with relapsed or refractory B cell precursor acute lymphoblastic leukemia (ALL).
**4. BREYANZI®** ***(lisocabtagene maraleucel)***<br><br>**FDA-approved in 2021[17]**<br><br>**Limitations of use:**	• Adult patients with large B cell lymphoma (LBCL), including diffuse large B cell lymphoma (DLBCL) not otherwise specified (including DLBCL arising from indolent lymphoma), high-grade B cell lymphoma, primary mediastinal large B cell lymphoma, and follicular lymphoma grade 3B, who have the following:<br>- Refractory disease to first-line chemoimmunotherapy or relapse within 12 months of first-line chemoimmunotherapy;<br>- Refractory disease to first-line chemoimmunotherapy or relapse after first-line chemoimmunotherapy and are not eligible for hematopoietic stem cell transplantation (HSCT) due to comorbidities or age;<br>- Relapsed or refractory disease after two or more lines of systemic therapy.<br><br>BREYANZI is **not** indicated for treatment of patients with primary central nervous system lymphoma.

II. B cell maturation antigen (BCMA)-directed genetically modified autologous T cell immunotherapies that have the following toxicities: cytokine release syndrome, neurological syndromes, macrophage activation syndrome (MAS), and hemophagocytic lymphohistiocytosis (HLH) and prolonged/recurrent cytopenia.

Product name and year it was approved	Indications and usage
**1. ABECMA®** ***(idecabtagene vicleucel)***<br><br>**FDA-approved in 2021[18]**	• Adult patients with relapsed or refractory multiple myeloma after four or more prior lines of therapy, including an immunomodulatory agent, a proteasome inhibitor, and an anti-CD38 monoclonal antibody.
**2. CARVYKTI®** ***(ciltacabtagene autoleucel)***<br><br>**FDA-approved in 2022[19]**	• Adult patients with relapsed or refractory multiple myeloma after four or more prior lines of therapy, including a proteasome inhibitor, an immunomodulatory agent, and an anti-CD38 monoclonal antibody.
